# Response to “Moghnieh et al. COVID-19: Second Wave or Multiple Peaks, Natural Herd Immunity or Vaccine – We Should be Prepared”

**DOI:** 10.1017/dmp.2020.456

**Published:** 2020-11-19

**Authors:** Dua Khalid, Haya Khalid, Syed Uzair Mahmood, Erum Choudry

**Affiliations:** 1 Department of Research, Jinnah Sindh Medical University, Karachi, Pakistan; 2 Medicine, Health and Life Sciences Department, Queen’s University Belfast, Northern Ireland, UK; 3 Indus Hospital Research Centre, The Indus Hospital, Karachi, Pakistan

**Keywords:** Pandemics, Public Health, Communicable Diseases

A recent article in your journal highlighted all the potential strategies to be adopted in case of the potential next wave of the pandemic.^1^ The article featured many mitigation and precautionary strategies that would possibly help control the situation in the future and minimize the risks faced due to certain practices during the global pandemic.

However, in our opinion, 1 more recommendation should have been incorporated in the article at the public health level. Another major issue that aggravated the situation during the pandemic was the inclination toward self-medication of the public. A study conducted in the Kingdom of Saudi Arabia showed that one-third of the respondents were in the favor of self-medication.^2^

Out of the many reasons, exaggeration on the news channels and social media triggers the public to be more inclined toward self-medication as an option. This can be proved by the data of Global Google trends where the search for chloroquine and hydroxychloroquine immediately spiked after the press conference addressed the potential benefits of these drugs for treating coronavirus disease (COVID-19).^3^ The potential beneficial outcomes grabbed people’s attention; however, an overdose of these drugs can lead to life-threatening outcomes and cannot go unnoticed.^4^ However, ivermectin, another drug believed to be a potential treatment, has its own side effects, and more than 2 doses cannot be administered. But the municipal head service of Natal recommended the regular use of ivermectin. Considering the easy availability of ivermectin without a prescription, self-medication and self-dosing could lead to the worsening of the function of the central nervous system, due to several drug-drug interactions.^5^

We believe that 1 mitigation strategy that could swiftly end this problem of self-medication is for the media to discontinue the hype on the usage of drugs and instead highlight the protocols for the administration of these drugs that by only trained professionals and health care workers. Light should be shed on the hazards of self-medication and self-dosing without following the precautions. Moreover, considering the fact that the protocols of all the drugs are easily available online and a “click away,” the public is more likely to not give them a read. Therefore, availability of the drugs without a prescription should be banned by the government and authorities. This would prove to be a more permanent and reliable solution to end the problem.
